# Fucoidan Induces Apoptosis of HT-29 Cells via the Activation of DR4 and Mitochondrial Pathway

**DOI:** 10.3390/md18040220

**Published:** 2020-04-20

**Authors:** Xu Bai, Yu Wang, Bo Hu, Qi Cao, Maochen Xing, Shuliang Song, Aiguo Ji

**Affiliations:** 1 Marine College, Shandong University, Weihai 264209, China; 15634407267@163.com (X.B.); wy392191187@163.com (Y.W.); hobophar@163.com (B.H.); sddxcqq@163.com (Q.C.); sddxxmc@163.com (M.X.); 2 School of Pharmaceutical Sciences, Shandong University, Jinan 250012, China

**Keywords:** fucoidan, apoptosis, DR4, mitochondrial pathway

## Abstract

Fucoidan has a variety of pharmacological activities, but the understanding of the mechanism of fucoidan-induced apoptosis of colorectal cancer cells remains limited. The results of the present study demonstrated that the JNK signaling pathway is involved in the activation of apoptosis in colorectal cancer-derived HT-29 cells, and fucoidan induces apoptosis by activation of the DR4 at the transcriptional and protein levels. The survival rate of HT-29 cells was approximately 40% in the presence of 800 μg/mL of fucoidan, but was increased to 70% after DR4 was silenced by siRNA. Additionally, fucoidan has been shown to reduce the mitochondrial membrane potential and destroy the integrity of mitochondrial membrane. In the presence of an inhibitor of cytochrome C inhibitor and DR4 siRNA or the presence of cytochrome C inhibitor only, the cell survival rate was significantly higher than when cells were treated with DR4 siRNA only. These data indicate that both the DR4 and the mitochondrial pathways contribute to fucoidan-induced apoptosis of HT-29 cells, and the extrinsic pathway is upstream of the intrinsic pathway. In conclusion, the current work identified the mechanism of fucoidan-induced apoptosis and provided a novel theoretical basis for the future development of clinical applications of fucoidan as a drug.

## 1. Introduction

**Figure 1:**
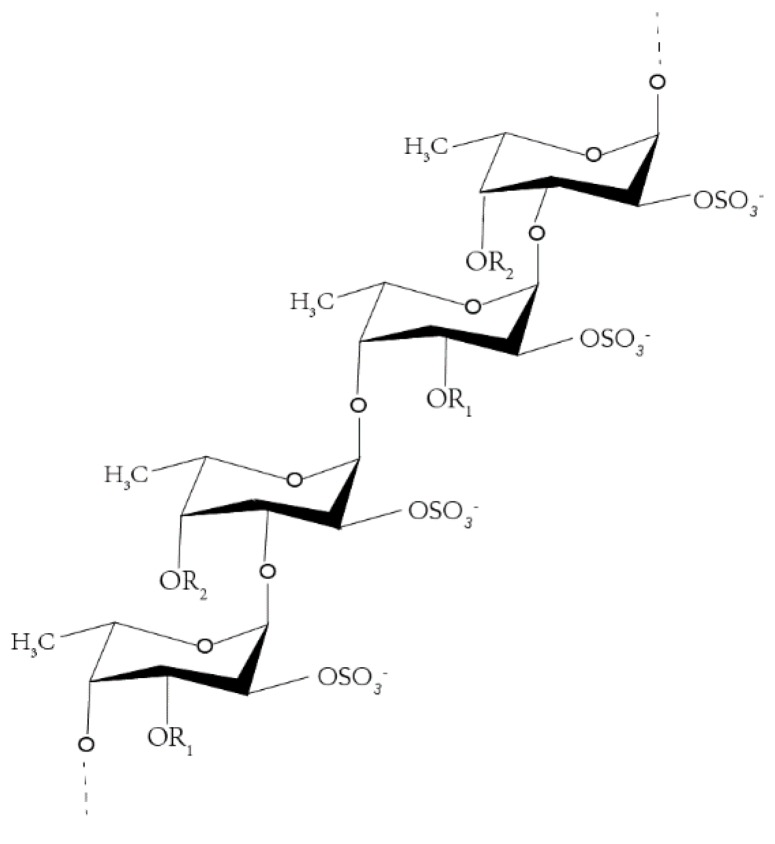
Fucoidan structure from *Fucus vesiculosus*.

Fucoidan is a water-soluble heteropolysaccharide, derived mostly from brown algae, such as *Fucus vesiculosus* (Figure 1) [1,2,3,4,5]. Recent studies have shown that the research on fucoidan mainly focuses on two aspects—one is to explore ways to increase the yield of fucoidan [6,7,8,9], while the other is to explore the various pharmacological activities of fucoidan [10,11,12], including anti-inflammatory [13,14], anti-tumor, anti-virus, hypolipidemic, antithrombotic, and so on [15], but less research exists on its mechanism. Owing to the characteristics of high incidence and high mortality of tumor, the prevention and treatment of tumor has become a global research trend. Fucoidan can exert anti-tumor effects mainly by inducing apoptosis [16,17], arresting cell cycle [18], inhibiting cell migration [18,19,20], and so on.

Studies have shown that fucoidan can induce apoptosis [16]. There are two main apoptotic pathways currently studied. One is the extrinsic pathway, the death receptor pathway, which activates apopain in cells through extrinsic signal transduction [21]; the TRAIL receptor directly recruits procaspase-8, which is activated to form caspase-8, and then activates downstream effector molecules. The other is the intrinsic pathway [21,22], the mitochondrial pathway, where external stimulation leads to the enhancement of mitochondrial outer membrane permeability and apoptosis-related proteins in the mitochondrial inner. Moreover, outer membrane spaces, such as cytochrome C, form apoptosome with apaf-1; activate cascades; and further activate caspase -3, -6, and -7. Both ways can activate the downstream effector molecule caspase, which may lead to the activation of nuclease and the degradation of important proteins [23]. If the activated caspase-8 is sufficient, caspase-3 will be activated directly to induce cell apoptosis through the receptor; if the activated caspase-8 is insufficient, caspase-8 will activate the mitochondrial pathway [24,25]. In this study, the receptor of fucoidan in the process of inducing HT-29 cell apoptosis was determined at the level of gene and protein, determining that the extrinsic pathway was involved in the process of cell apoptosis; at the same time, it was found that fucoidan could affect the mitochondrial membrane potential and induce cell apoptosis through the mitochondrial pathway.

Apoptosis is usually mediated by a variety of signaling pathways, including NF-kB, PI3K, JNK, and so on [26,27,28,29]. The activation of JNK is stress-induced and plays an important role in the process including cell proliferation, differentiation, and tumor transformation.

In this study, we found that fucoidan can affect the migration, cycle, and apoptosis of HT-29 cells, and the effect of inducing apoptosis of HT-29 cells was the most significant. Thus, the purpose of this study was to explore the receptor and mechanism of fucoidan-induced apoptosis in HT-29 cells preliminarily. On one hand, it can lay a theoretical foundation for the application of fucoidan in dietary supplements and drugs; on the other hand, it can provide research support for the high-value development of kelp resources.

## 2. Results

### 2.1. Cytotoxicity of Fucoidan

**Figure 2:**
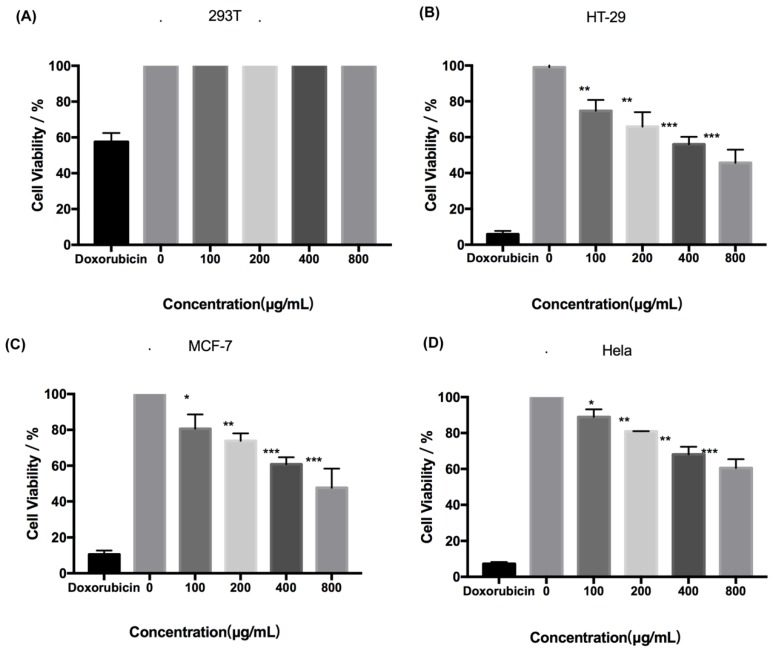
Cytotoxicity of fucoidan. (**A**) Toxicity of fucoidan to 293T cell is expressed as the means ± SD (n = 3). (**B**) Toxicity of fucoidan to HT-29 cells is expressed as the means ± SD (n = 3). (**C**) Toxicity of fucoidan to MCF-7 cell is expressed as the means ± SD (n = 3). (**D**) Toxicity of fucoidan to HeLa cell is expressed as the means ± SD (n = 3). *, *p* < 0.05; **, *p* < 0.01; ***, *p* < 0.001.

Fucoidan administered at concentrations up to 800 μg/mL was not cytotoxic to human normal cell 293T cells (Figure 2A). However, fucoidan induced death of HeLa, MCF-7, and HT-29 cells in a dose-dependent manner (Figure 2B–D). The cytotoxic effect of fucoidan was most pronounced in HT-29 cells, with the cell survival rate of only approximately 40% at 800 μg/mL of the compound. Therefore, HT-29 cells and fucoidan concentrations of up to 800 μg/mL were selected for further experiments.

### 2.2. Pharmacological Activity of Fucoidan on HT-29 Cells

**Figure 3:**
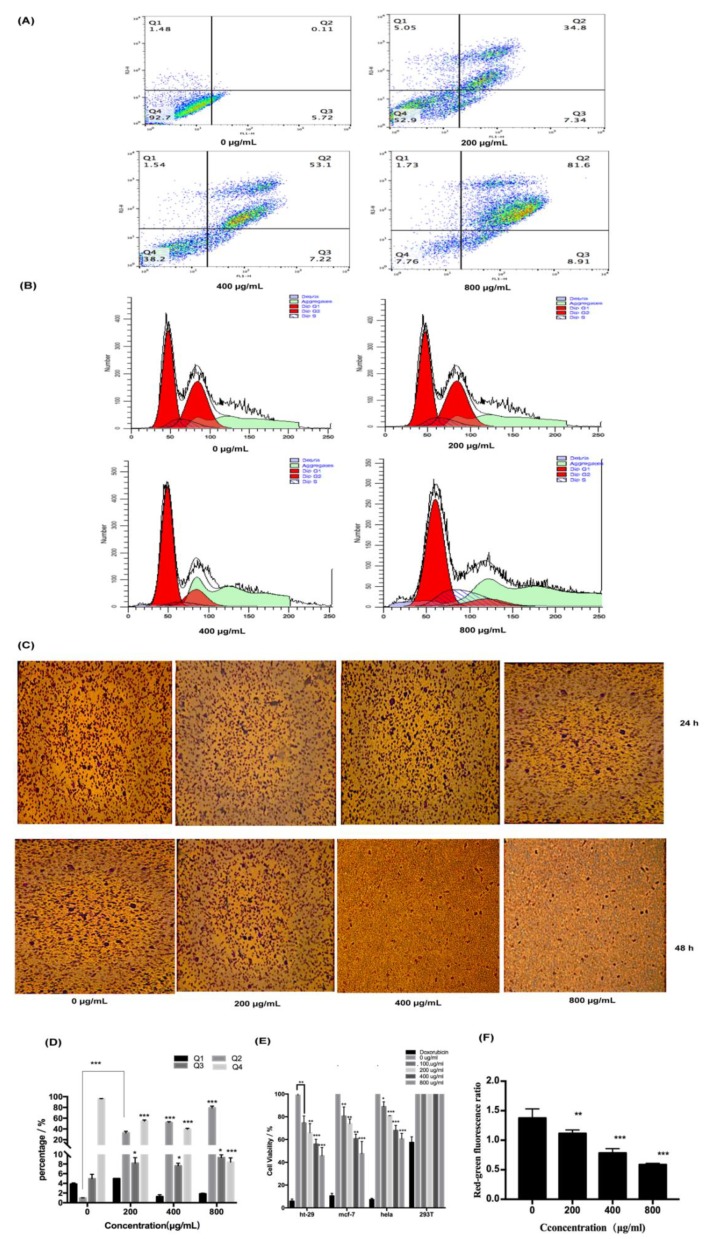
Pharmacological activity of fucoidan on cells. (**A**) Detection of apoptosis by flow cytometry. (**B**) Detection of cell cycle by flow cytometry. (**C**) Detection of cell migration. (**D**) Statistical results of apoptosis are expressed as the means ± SD (n = 3). (**E**) Statistical results of cell cycle are expressed as the means ± SD (n = 3). (**F**) Statistical results of cell migration are expressed as the means ± SD (n = 3). *, *p* < 0.05; **, *p* < 0.01; ***, *p* < 0.001.

To explore the pharmacological effects of fucoidan on HT-29 cells, apoptosis, migration, and cell cycle were analyzed. We can find that the treatment increased the rate of apoptosis of HT-29 cells in a dose-dependent fashion, with 80% of the cells in the late stage of apoptosis at 800 μg/mL of fucoidan (Figure 3A,D). However, fucoidan blocked the cells in the G0/G1 phase of the cell cycle, with 50% of the cells in the G0/G1 phase of the cell cycle at 800 μg/mL of fucoidan, and the fraction of arrested cells increased with higher fucoidan concentrations (Figure 3B,E). Additionally, the migration of HT-29 cells tended to decrease with increasing fucoidan concentration and incubation time, but the reduction in migratory activity did not reach statistical significance, remaining at approximately 30% at 800 μg/mL (Figure 3C,F). These findings indicated that fucoidan affected apoptosis more significantly than migration and cell cycle.

### 2.3. Analysis of Fucoidan-Induced Apoptosis of HT-29 Cells

#### 2.3.1. Fucoidan Can Induce Apoptosis Through the Extrinsic Pathway

**Figure 4:**
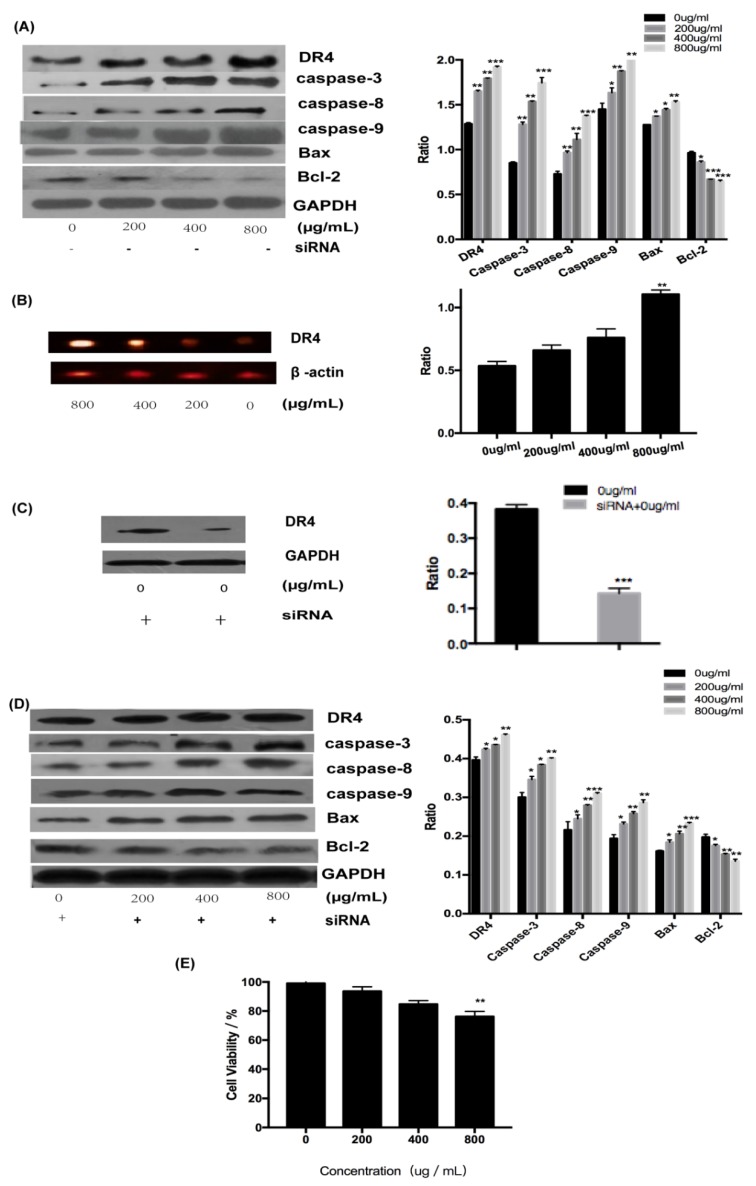
Fucoidan induced apoptosis through DR4. (**A**) Results of Western blotting of proteins. (**B**) Results of Reverse Transcription-Polymerase Chain Reaction (RT-PCR) with DR4. (**C**) Results of Western blotting of proteins with the silent DR4. (**D**) Expression of proteins after DR4 was silenced. (**E**) The toxicity of fucoidan to HT-29 cells with silent DR4 is expressed as the means ± SD (n = 3). *, *p* < 0.05; **, *p* < 0.01; ***, *p* < 0.001.

To explore the involvement of receptors in the activation of apoptosis by fucoidan, the expression of DR4 and related proteins at the transcriptional and translational level was determined. All examined proteins, including DR4 and caspase-3, -6, and -9, were upregulated by fucoidan in a concentration-dependent manner (Figure 4A). The expression level of DR4 increased with the increase of fucoidan concentration at the gene level and the result demonstrated that DR4 was required for the induction of apoptosis by fucoidan (Figure 4B). To determine whether DR4 was required for the induction of apoptosis by fucoidan, siRNA was used to silence its expression, whose silence rate was about 65% (Figure 4C). However, although the expression of all examined proteins was suppressed in the presence of siRNA targeting DR4 (Figure 4D), these proteins did not decrease significantly with the increasing concentration in comparison, which may be because of DR4’s low silence rate. However, DR4 silencing decreased the cytotoxicity of fucoidan (800 μg/mL) on HT-29 cells, resulting in an increase in the survival rate from 40% to 75% (Figure 4E). These results demonstrated that fucoidan can induce apoptosis of HT-29 cells by upregulating DR4.

#### 2.3.2. Fucoidan Can Induce Apoptosis Through the Intrinsic Pathway

**Figure 5:**
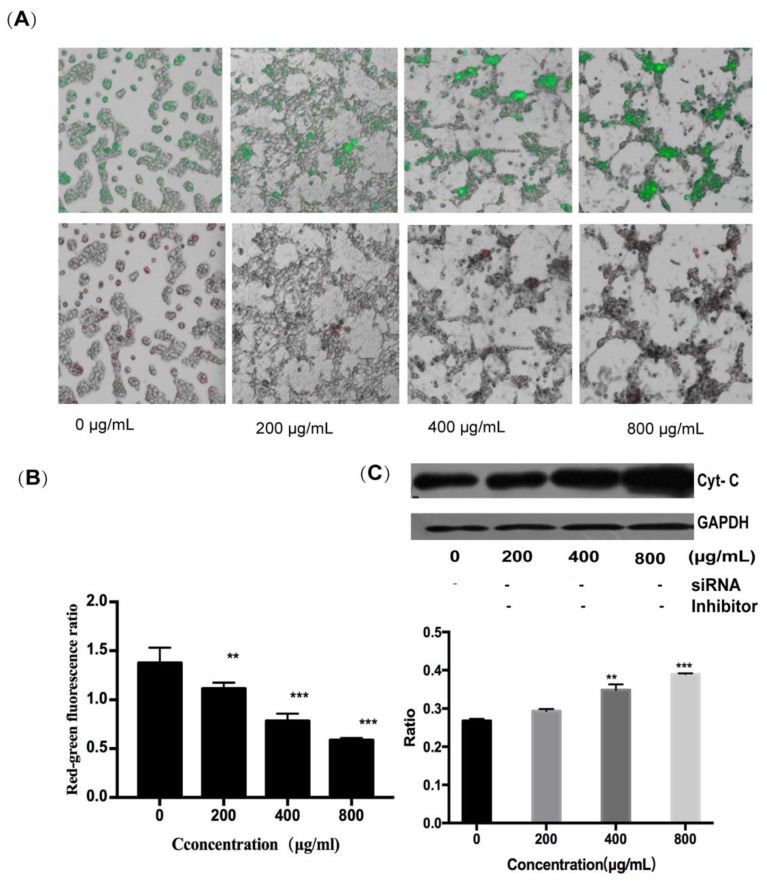
Changes of membrane potential induced by fucoidan in HT-29 cells. (**A**) Results of JC-1 staining. (**B**) Results of red-green fluorescence ratio. (**C**) Results of Western blotting of cytochrome C. **, *p* < 0.01; ***, *p* < 0.001.

To determine whether the mitochondrial pathway can contribute to fucoidan-induced apoptosis of HT-29 cells, the changes in mitochondrial membrane potential were determined by the JC-1 probe, and the expression of cytochrome C protein was assessed by Western blotting. It can be seen that, with an increase in fucoidan concentration, the red-to-green ratio of JC-1 fluorescence decreased from 1.3 to 0.6, indicating a reduction in the inner mitochondrial membrane potential and an increase in its permeability (Figure 5A,B). Concurrently, cytochrome C was released from the mitochondria into the cytoplasm; this effect was also dependent on fucoidan concentration, and the ratio of cytochrome C expression in the experimental group to the control group was 1.56 at 800 μg/mL (Figure 5C). The release of cytochrome C initiated the caspase cascade, leading to apoptosis. These results showed that fucoidan activated not only the extrinsic pathway through surface death receptors, but also the intrinsic mitochondrial pathway-mediated apoptosis of HT-29 cells.

#### 2.3.3. Relationship Between the Extrinsic and Intrinsic Pathways

**Figure 6:**
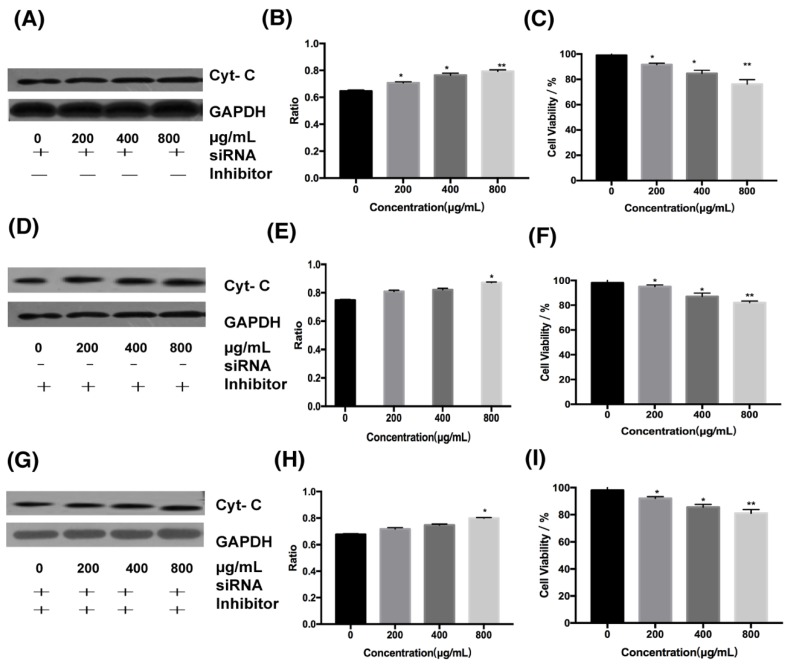
Effects of cytochrome C inhibitor on HT-29 cells. (**A**) Results of Western blotting of cytochrome C with the silent DR4. (**B**) Results of Western blotting of cytochrome C with cytochrome C inhibitor. (**C**) Results of Western blotting of cytochrome C with cytochrome C inhibitor and silent DR4. (**D**) Results of protein expression ratio at different concentrations with the silent DR4. (**E**) Results of protein expression ratio at different concentrations with cytochrome C inhibitor. (**F**) Results of protein expression ratio at different concentrations with cytochrome C inhibitor and silent DR4. (**G**) The toxicity of fucoidan to HT-29 cells with the silent DR4 is expressed as the means ± SD (n = 3). (**H**) The toxicity of fucoidan to HT-29 cells with cytochrome C inhibitor is expressed as the means ± SD (n = 3). (**I**) The toxicity of fucoidan to HT-29 cells with cytochrome C inhibitor and silent DR4 is expressed as the means ± SD (n = 3). *, *p* < 0.05; **, *p* < 0.01.

Cells in this experiment were divided into three groups: the first group, cells treated with siRNA for DR4; the second group, cells treated with cytochrome C inhibitor; and the third group, cells treated simultaneously with cytochrome C inhibitor and DR4 siRNA. We can find that in cells treated only with DR4 siRNA at 800 μg/mL, the ratio of cytochrome C expression in the experimental group to the control group decreased from 1.56 to 1.32 (Figure 5C and Figure 6A,B), and the cell survival rate was increased from 40% to about 75%( Figure 2B and Figure 6C). When cells were treated only with the inhibitor of cytochrome C or treated simultaneously with cytochrome C inhibitor and DR4 siRNA, there was no significant difference in the ratio and cell survival rate between the second and third group. In the second and third group, it can be seen that the ratio decreased from 1.56 to 1.15 and 1.13 at 800 μg/mL, respectively (Figure 5C and Figure 6D–H), both lower than the ratio after inhibiting the death receptor pathway; besides, at 800 μg/mL, the cell survival rate was increased from 40% to about 80% ( Figure 2B and Figure 6F,I), both higher than 75%.Therefore, the difference of cell survival rate and cytochrome C expression indicated that the mitochondrial pathway was downstream of the DR4 pathway.

### 2.4. Effect of Fucoidan on the JNK Signaling Pathway in HT-29 Cells

**Figure 7:**
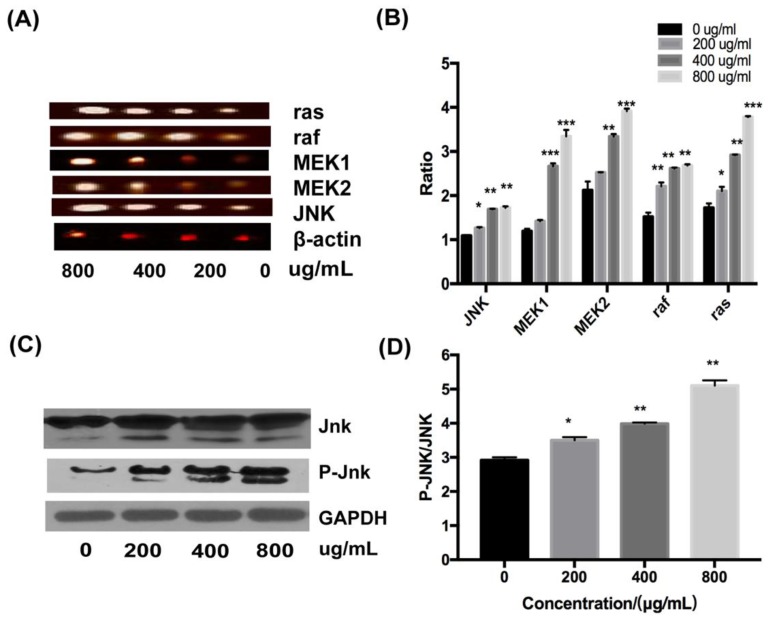
Effects of fucoidan on JNK signaling pathway in HT-29 cells. (**A**) Results of RT-PCR. (**B**) Ratio of related expression factors at mRNA level. (**C**) Results of Western blotting of related proteins. (**D**) Ratio of JNK to p-JNK. *, *p* < 0.05; **, *p* < 0.01; ***, *p* < 0.001.

The role of the JNK signal pathway in the induction of apoptosis in HT-29 cells was determined by RT-PCR and Western blotting. At the mRNA level, the expression of ras, raf, MEK1, MEK2, and JNK were upregulated with increasing concentrations of fucoidan (Figure 7A,B). Moreover, fucoidan increased the level of JNK protein and its phosphorylated form, P-JNK, in a dose-dependent fashion, as assessed by Western blot analysis (Figure 7C); the ratio of p-JNK/JNK increased from 2.9 to about 5.2 at 800 μg/mL (Figure 7D). Therefore, the JNK signaling pathway was essential for the activation of apoptosis of HT-29 cells by fucoidan.

## 3. Discussion

Cancer is the leading cause of death worldwide, and colorectal cancer has become a common type of cancer. Given the high incidence, morbidity, and mortality of colorectal cancer, significant research effort is focused on its prevention and treatment. The current work documented that fucoidan affects the migration, apoptosis, and cell cycle progression of colorectal cancer-derived HT-29 cells. Among these effects, the induction of apoptosis was the most potent one. Further analysis of the mechanisms implicated in triggering apoptosis indicated that fucoidan induced this process in HT-29 cells through simultaneous activation of the intrinsic and extrinsic pathways by the JNK signaling pathway.

In the presence of 800 μg/mL of fucoidan, the cell survival rate was approximately 40%, and the induction of apoptosis in HT-29 cells was accompanied by the increase in expression of DR4 at the mRNA and protein levels. After silencing DR4 with siRNA, the difference in the expression of DR4 gradually decreased after inhibiting the extrinsic pathway. Although the expression of the protein was not significantly different from that when DR4 is not silenced, the fraction of surviving cells increased from 40% to 75% at 800 μg/mL of fucoidan, further documenting the critical role DR4 played in the apoptosis of HT-29 cells. In addition, the red-to-green ratio of JC-1 fluorescence decreased from 1.3 to 0.6, suggesting that fucoidan can destroy mitochondrial membrane integrity. These changes triggered the intrinsic apoptotic pathway by the release of cytochrome C into the cytoplasm.

To explore the relationship between the intrinsic and extrinsic pathways, inhibitor of cytochrome C inhibitors and siRNA targeting DR4 siRNA were added. In this experiment, at 800 μg/mL, the cell survival rate was increased from 40% to about 75% when cells were treated only with DR4 siRNA. When the intrinsic pathway or both pathways were inhibited simultaneously, the cell survival rates were improved without significant differences, both from 40% to about 80%, which were higher than the cell survival rates after inhibiting the extrinsic pathway. In addition, the difference in the expression of cytochrome C gradually decreased after inhibiting the extrinsic pathway; the ratio of the expression of cytochrome C in the experimental group to that of the control group decreased from 1.56 to 1.32 at the concentration of 800 μg/mL. When the intrinsic pathway or both pathways were inhibited simultaneously, the difference in cytochrome C expression was significantly reduced without significant differences, and the ratio decreased from 1.56 to 1.15 and 1.13, respectively, at 800 μg/mL, both lower than the ratio after inhibiting the extrinsic pathway. Therefore, the ratios of cytochrome C expression and cell survival after inhibiting the intrinsic pathway were similar to those after inhibiting both pathways, which were different from those after inhibiting the extrinsic pathway, indicating that the intrinsic pathway was in the downstream of the extrinsic pathway.

It was established that fucoidan inhibited the proliferation activity of HT-29 cells through simultaneous activation of the intrinsic and extrinsic pathways by the JNK signaling pathway, and the intrinsic pathway was downstream of the extrinsic pathway. However, there was still much controversy about whether the intrinsic and extrinsic pathways played an important role in inducing apoptosis in HT-29 cells owing to the lack of untransfected control within the same experiment, which must be improved in order to get more convincing results. In addition, a study showed that the cell survival rate was only approximately 80% with cells treated with cytochrome C inhibitor or with cytochrome C inhibitor and DR4 siRNA simultaneously; not all cells survived. On one hand, the silencing of DR4 or inhibition of the expression of cytochrome C could not be completed; on the other hand, fucoidan can play other roles besides inducing apoptosis, such as fucoidan significantly blocking the cell cycle, while inducing apoptosis with the increase in concentration. Therefore, future studies should address the impact of fucoidan on both apoptosis and cycle of HT-29 cells. These studies may reveal the inevitable connection between cell cycle and apoptosis and further clarify the mechanism of the induction of HT-29 cell death by fucoidan.

## 4. Materials and Methods 

### 4.1. Materials

HT-29, MCF-7, Hela, and 293T cells were purchased from Kunming Institute of Zoology, Chinese Academy of Sciences Kunming Cell Bank; Dulbecco’s minimum essential medium (DMEM) was purchased from GE Healthcare HyClone, USA; trypsin was purchased from Invitrogen, US; penicillin and streptomycin were purchased from Invitrogn, USA; Methylthiazolyldiphenyl-tetrazolium bromide (MTT) was purchased from Beyotime Biotechnology, China; Ddimethylsulphoxide (DMSO) was purchased from Sigma-Aldrich, USA; fucoidan from *F. vesiculosus* was provided by Shandong University, where the average molecular weight of fucoidan is about 1300 kDa, as analyzed by size exclusion chromatography (fucose content is about 80%, galactose is about 9%, glucuronic acid is about 7%, glucose is about 2%, xylose is about 1%, and rhamnose is about 1%, which were detected by high performance liquid chromatography (HPLC) after acid hydrolysis) [30,31]; Cytochrome C inhibitor was purchased from abcam, UK; cell cycle and apoptosis analysis kit was purchased from Beyotime, China; mitochondrial membrane potential assay kit with JC-1 was purchased from Beyotime, China; Transwell chamber (8.0 μm) was purchased from corning, USA; primary antibodies against Bcl-2 (product number: AB112), BAX (product number: AB026), P-JNK/SAPK (product number: AJ516), JNK/SAPK (product number: AJ518), caspase-8 (product number: AC056), caspase-9 (product number: AC062), GAPDH (product number: AG019), and cytochrome C (product number: AC909) were purchased from Beyotime, China; primary antibodies against DR4 (product number: 24063-1-AP) and caspase-3 (product number: 66470-2-Ig) were purchased from Proteintech, USA; horseradish peroxidase-conjugated anti-rabbit (product number: A0208) and anti-mouse (product number: A0216) secondary antibodies were purchased from Beyotime, China; diluent was purchased from Beyotime, China; siRNA (DR4) was purchased from Sangon Biotech, China; Lipo8000 transfection reagent was purchased from Beyotime, China; PCR primer was purchased from Sangon Biotech, China; revert aid first strand cDNA synthesis kit was purchased from ThermoFisher, USA; and dream taq green PCR master mix (2X) was purchased from ThermoFisher, USA.

### 4.2. Cell Culture 

Colon cancer cell line (HT-29), breast cancer cell line (MCF-7), cervical cancer cell line (HeLa), and renal epithelial cell line (293T) were cultured in DMEM medium supplemented with 20% (v/v) fetal bovine serum (FBS) and 1% (v/v) antibiotics. The cells were incubated in a 5% CO_2_ incubator at 37 °C. 

### 4.3. MTT Assay for Cell Viability 

The cytotoxicity of fucoidan toward HT-29, Hela, MCF-7, and 293T cells was determined using the MTT assay. The cells were seeded in a 96-well plate (6 × 10^3^ cells/well), and fucoidan (0, 100, 200, 400, and 800 μg/mL) was added after 24 h, Doxorubicin, 5 μg/mL, was used as a positive control. After incubation for 48 h, a 10 μL aliquot of MTT solution (5 mg/mL) was added to each well, and the cells were incubated for an additional 4 h. The medium was then aspirated, 100 μL of DMSO was added to each well, and the plates were vortexed for 3 min until thoroughly mixed. The absorbance of each well was measured at a wavelength of 490 nm using a microplate reader (Sunrise, Tecan, Austria). 

### 4.4. Cell Cycle Analysis 

After treatment with fucoidan, the cells were harvested with trypsin and washed twice with cold phosphate buffer saline (PBS). Subsequently, the cell nuclei were stained using the cell cycle and apoptosis analysis kit following the manufacturer’s instructions. The cells were analyzed by flow cytometry and the relative DNA content was determined using the Modfit software (5.0.9., Verity, Topsham, VT, USA).

### 4.5. Cell Migration Assay 

For cell migration assay, HT-29 cells were grown to 80% confluence, harvested, and seeded in 24-well Transwell plates with 8 μm pores at the density of 5 × 10^4^ cells/well. DMEM containing 10% FBS was added to the lower chamber. After 24 h, 0, 200, 400, and 800 μg/mL of fucoidan was added to the upper chamber, and the cells were cultured for 24 h and 48 h, respectively. At the end of incubation, cells were stained with crystal violet, photographed, and counted under an inverted microscope (DM100FL, Leica, Germany).

### 4.6. Quantification of Apoptosis with the Annexin V/Propidium Iodide Assay 

Cells in log-phase growth were seeded in six-well plates at a density of 1 × 10^5^ cells/well. After 24 h, 0, 200, 400, and 800 μg/mL of fucoidan was added and the cultures were incubated for 48 h. The cells were then harvested, washed, and resuspended in the binding buffer containing Annexin V and propidium iodide (PI). After incubation for 15 min at room temperature, the stained cells were analyzed by flow cytometry. Data were analyzed using FlowJo software (10.0.7., BD, Franklin, NJ, USA).

### 4.7. Total RNA Extraction and Reverse Transcription-Polymerase Chain Reaction

RNA was isolated and reverse-transcribed into cDNA using the revert aid first strand cDNA synthesis kit, according to the manufacturer’s protocol. For PCR, the cDNA was mixed with forward and reverse primers (for the list of primers, see Table 1) and dream taq green PCR master mix (2x). The amplification included 30 cycles of denaturation for 30 s at 95 °C, annealing for 30 s at 57 °C, and elongation for 30 s at 72 °C. The PCR products were subjected to electrophoresis on 1% agarose gel. The gel was analyzed using the Image J software, and the amount of cDNA was determined and normalized to the amount of β-actin cDNA. 

### 4.8. Silencing the Expression of DR4 by siRNA

The cells in the logarithmic phase of growth were seeded in a six-well plate at a density of 2 × 105 cells/well. After 24 h, a mixture of 6 μL Lipo 8000 and 150 μL siRNA (sense: 5’-GCUGUUCUUUGACAAGUUUTT3’, antisense: 5’ AAACUUGUCAAAGAACAGCTT-3’) was added, and the medium was replenished to 2 mL. The mixture was aspirated after 6 h, and the cells were incubated with 0, 200, 400, and 800 μg/mL of fucoidan for an additional 48 h.

### 4.9. Detection of Mitochondrial Membrane Potential by JC-1 Staining

The cells in the logarithmic phase of growth were seeded in a six-well plate at a density of 6 × 10^3^ cells/well. After 24 h, 0, 200, 400, and 800 μg/mL of fucoidan were added. The cells were stained 48 h later using the mitochondrial membrane potential detecting kit (JC-1); the manufacturer’s protocol was employed. Stained cells were photographed and counted using a multi-function microplate reader.

### 4.10. Inhibition of Cytochrome C Expression

The cells in the logarithmic phase of growth were seeded in a six-well plate at a density of 6 × 10^3^ cells/well. Then, 3 μM cytochrome C inhibitor was added after 24 h. One hour later, the inhibitor was washed out, and the cells were incubated for an additional 48 h in the presence of 0, 200, 400, and 800 μg/mL of fucoidan.

### 4.11. Western Blot Analysis 

Cells were lysed with RIPA lysis buffer containing a phosphatase inhibitor and PMSF. Extracted proteins were subjected to electrophoresis on 10% SDS-polyacrylamide gel and transferred to a PVDF membrane. The membrane was blocked with 5% (w/v) skim milk powder in TBST for 1 h, and incubated with primary antibodies at 4 °C overnight, according to the manufacturer’s protocol (dilution ratio of all proteins was 1:1000, except for cytochrome c, which was 1:200). After washing, membranes were incubated with peroxidase-conjugated secondary antibodies (dilution ratio was 1:1000), and protein bands were visualized using the hypersensitive enhanced chemiluminescence (ECL) chemiluminescence kit. Densitometric analysis was performed using the Image J software (1.8.0, NIH, US), and the intensity of specific bands was normalized to the GAPDH band.

### 4.12. Statistical Analysis

All data in graphs were presented as the mean value ± standard deviation from three independent measurements. The statistical analysis was used in statistical software (SPSS, Chicago, IL, USA) and GraphPad Prism 7.00 (GraphPad Software, CA, USA). *p* < 0.05 was considered significant. 

## Figures and Tables

**Table 1:** Primer sequence table.

	Primers	Sequences	Product Size
Ras	Forward	5’-CGACACAGCAGGTCAAGAGG-3’	20
	Reverse	5’-GGCATCATCAACACCCTGTCT-3’	21
Raf	Forward	5’-CAGCGAATCAGCCTCACCTTCAG-3’	23
	Reverse	5’-CGCAGAACAGCCACCTCATTCC-3’	22
β-actin	Forward	5’-CGTGGACATCCGCAAAGAC-3’	19
	Reverse	5’-GCATTTGCGGTGGACGAT-3’	18
DR4	Forward	5’-CTGATCACCCAACAAGACCTAG-3’	22
	Reverse	5’-GATGCAATCTCTACCGCTTCT-3’	22
JNK	Forward	5’-GGAATGGCCTGCCTTACGATGAC-3’	23
	Reverse	5’-GGCTCTGTTGCTGCCACTGC-3’	20
MEK1	Forward	5’-CAGCTCTGCGGAGACCAACTTG-3’	22
	Reverse	5’-CTGATCTCGCCATCGCTGTAGAAC-3’	24
MEK2	Forward	5’-ACTTGACGAGCAGCAGAAGAAGC-3’	23
	Reverse	5’-GAGCCGCCGTCCATGTGTTC-3’	20
